# The ClinGen Syndromic Disorders Gene Curation Expert Panel: Assessing the clinical validity of 111 gene-disease relationships

**DOI:** 10.1016/j.gimo.2025.103429

**Published:** 2025-04-09

**Authors:** Eleanor C. Broeren, Vanessa N. Gitau, Alicia B. Byrne, Pamela Ajuyah, Marie B. Balzotti, Jonathan S. Berg, Krista Bluske, B. Monica Bowen, Matthew P. Brown, Amanda Buchanan, Brendan T. Burns, Nicole J. Burns, Anjana Chandrasekhar, Aditi Chawla, Jessica X. Chong, Maya Chopra, Amanda R. Clause, Marina T. DiStefano, Stephanie DiTroia, Marwa A. Elnagheeb, Amanda N. Girod, Himanshu Goel, Katie L. Golden-Grant, Thuong Ha, Ada Hamosh, Jennifer M. Huang, Madeline Y. Hughes, Saumya S. Jamuar, Sylvia Kam, Akanchha Kesari, Ai Ling Koh, Rhonda N.T. Lassiter, Sarah E. Leigh, Gabrielle Lemire, Jiin Ying Lim, Alka Malhotra, Hannah R. McCurry, Becky Milewski, Shahida Moosa, Stephen A. Murray, Emma H. Owens, Elizabeth E. Palmer, Brooke C. Palus, Mayher J. Patel, Revathi Rajkumar, Julie C. Ratliff, F. Lucy Raymond, Bruno Della Ripa Rodrigues Assis, Samin A. Sajan, Zinayida Schlachetzki, Sarah A. Schmidt, Zornitza Stark, Samuel P. Strom, Julie P. Taylor, Courtney Thaxton, Devon L. Thrush, Sabrina Toro, Kezang C. Tshering, Nicole A. Vasilevsky, Bess Wayburn, Ryan F. Webb, Anne O’Donnell-Luria, Alison J. Coffey

**Affiliations:** 1 Program in Medical and Population Genetics, Broad Institute of MIT and Harvard, Cambridge, MA; 2 Myriad Women’s Health, Myriad Genetics, South San Francisco, CA; 3 Genetics, The University of North Carolina at Chapel Hill, Chapel Hill, NC; 4 Illumina Laboratory Services, Illumina Inc, San Diego, CA; 5 Ambry Genetics, Aliso Viejo, CA; 6 Biomedical Data Science, Stanford University, Palo Alto, CA; 7 Maternal-Fetal Medicine, Tufts Medical Center, Boston, MA; 8 Department of Pathology and Laboratory Medicine, Children’s Hospital Colorado, Aurora, CO; 9 Pediatrics, University of Washington, Seattle, WA; 10 Brotman-Baty Institute for Precision Medicine, Seattle, WA; 11 Rosamund Stone Zander Translational Neuroscience Center, Boston Children’s Hospital, Boston, MA; 12 Neurology, Washington University in St. Louis, St. Louis, MO; 13 Hunter Genetics, Waratah, NSW, Australia; 14 Rady Children’s Institute for Genomic Medicine, San Diego, CA; 15 Genetics and Molecular Pathology, SA Pathology, Adelaide, SA, Australia; 16 Alliance between SA Pathology and UniSA, Centre for Cancer Biology, Adelaide, SA, Australia; 17 Adelaide Medical School, University of Adelaide, Adelaide, SA, Australia; 18 Department of Genetic Medicine, Johns Hopkins University School of Medicine, Baltimore, MD; 19 Genetics Service, Department of Paediatric Medicine, KK Women’s and Children’s Hospital, Singapore; 20 SingHealth Duke-NUS Genomic Medicine Centre, Singapore; 21 SingHealth Duke-NUS Institute of Precision Medicine, Singapore; 22 Biocuration, Genomics England Ltd, London, United Kingdom; 23 Division of Genetics and Genomics, Boston Children’s Hospital, Boston, MA; 24 Children’s Hospital of Eastern Ontario, Ottawa, ON, Canada; 25 Hereditary Cancer Genetic Counselor, PreventionGenetics part of Exact Sciences, Marshfield, WI; 26 Division of Molecular Biology and Human Genetics, Stellenbosch University, Cape Town, WC, South Africa; 27 Medical Genetics, Tygerberg Hospital, Cape Town, WC, South Africa; 28 The Jackson Laboratory, Bar Harbor, ME; 29 School of Paediatrics and Child Health, Faculty of Medicine and Health, University of New South Wales, Randwick, NSW, Australia; 30 Department of Medical Genetics, University of Cambridge, Cambridge, United Kingdom; 31 Neurogenetics, University of São Paulo, São Paulo, SP, Brazil; 32 Cytogenetics, Wisconsin State Laboratory of Hygiene, University of Wisconsin-Madison, Madison, WI; 33 Pediatrics, University of Wisconsin School of Medicine and Public Health, Madison, WI; 34 Alzheimer’s Therapeutic Research Institute (ATRI), University of Southern California, San Diego, CA; 35 Clinical Genomics Research and Development, Natera, Inc, Austin, TX; 36 Australian Genomics, Melbourne, VIC, Australia; 37 Victorian Clinical Genetics Services, Murdoch Children’s Research Institute, Melbourne, VIC, Australia; 38 Department of Paediatrics, University of Melbourne, Melbourne, VIC, Australia; 39 Clinical Services, Fabric Genomics, Oakland, CA; 40 Data Collaboration Center, Critical Path Institute, Tucson, AZ; 41 Translational Research, Illumina Inc, Cambridge, United Kingdom

**Keywords:** ClinGen, Gene curation, Gene-disease relationship, Rare disease, Syndromic disorders

## Abstract

**Purpose:**

The Clinical Genome Resource (ClinGen) Gene Curation Expert Panels have historically focused on specific organ systems or phenotypes; thus, the ClinGen Syndromic Disorders Gene Curation Expert Panel (SD-GCEP) was formed to address an unmet need.

**Methods:**

The SD-GCEP applied ClinGen’s framework to evaluate the clinical validity of genes associated with rare syndromic disorders. A total of 111 gene-disease relationships (GDRs) associated with 100 genes spanning the clinical spectrum of syndromic disorders were curated.

**Results:**

From April 2020 through March 2024, 38 precurations were performed on genes with multiple disease relationships and were reviewed to determine if the disorders were part of a spectrum or distinct entities. A total of 14 genes were lumped into a single disease entity, and 24 were split into separate entities, of which 11 were curated by the SD-GCEP. A full review of 111 GDRs for 100 genes followed, with 78 classified as Definitive, 9 as Strong, 15 as Moderate, and 9 as Limited, highlighting cases in which further data are needed. All diseases involved 2 or more organ systems, whereas the majority (88/111 GDRs, 79.2%) had 5 or more organ systems affected.

**Conclusion:**

The SD-GCEP addresses a critical gap in gene curation efforts, enabling inclusion of genes for syndromic disorders in clinical testing and contributing to keeping pace with the rapid discovery of new genetic syndromes.

## Introduction

Syndromic disorders are complex conditions involving multiple organs or body systems and are highly enriched among infants with structural birth defects. Combined, genetic disorders and congenital malformations represent the leading cause of infant mortality in the United States, responsible for 20% of deaths^1^^,^^2^ and have a large health economic burden in children and adults.^3^ Syndromic disorders are one of the largest and fastest growing categories of disorders with multiple new syndromes published every month.^4^ To ensure the clinical validity of genetic testing for syndromic disorders, it is critical to rigorously evaluate the underlying evidence and classify the validity of purported gene-disease relationships (GDRs).

GDR validity curation is an essential first step for accurate and consistent clinical interpretation across variant triage, classification, and reporting.^5^ The American College of Medical Genetics and Genomics recommends that diagnostic gene panels include only GDRs that meet the Clinical Genome Resource’s (ClinGen) criteria for Definitive, Strong, or Moderate evidence. GDRs with a classification of Limited should typically only be assessed as part of exploratory exome or genome analysis, whereas GDRs classified as Disputed or Refuted are not appropriate for diagnostic testing.^6^ For variant classification,^7^ variants found in genes with only a Moderate GDR should not exceed a classification of likely pathogenic, and variants in genes with Limited evidence should not be classified above a variant of uncertain significance.^6^

ClinGen, a National Institutes of Health National Human Genome Research Institute-funded initiative, is building an authoritative central resource to define the clinical relevance of genes and variants for use in precision medicine and research.^8^ To achieve this goal, ClinGen has developed an evidence-based gene-disease validity curation framework that allows semiquantitative assessment of the strength of evidence underlying GDRs, which is then translated into 7 qualitative classifications. Four classification categories indicate evidence supporting a GDR (Definitive, Strong, Moderate, and Limited), whereas 2 categories indicate contradictory evidence (Disputed, Refuted). A Strong classification has the same level of evidence as a Definitive classification, but less than 3 years have passed between publications documenting human genetic evidence. No Known Disease Relationship indicates the GDR is not supported by human genetic evidence. Curated evidence is reviewed by gene curation expert panels (GCEPs), with appropriate clinical and laboratory expertise, and the resultant classification confirmed or adjusted based on expert insight.^9^

ClinGen GCEPs have historically focused on a specific organ system or phenotype. The challenge of the growing number of syndromic disorders published each year not covered by these curation efforts provided an opportunity for a GCEP to take a different approach to address this unmet need. The ClinGen Syndromic Disorders GCEP (SD-GCEP; https://clinicalgenome.org/affiliation/40060) was therefore established in March 2020 to classify the clinical validity of GDRs involving multiple body systems not under evaluation by another GCEP and is cofunded by the National Institute of Child Health and Human Development and National Institute of Neurological Disorders and Stroke. The SD-GCEP currently comprises 46 expert and biocurator members (and 25 former members) across 41 institutions and 6 continents. The GCEP meets twice a month plus additional quarterly meetings to accommodate as many time zones as possible. On average, 20 members representing 10 different institutions attend each call. Attendance ranges from 9 to 35 members including at least 1 but typically both of the chairs. Here, we describe the work of the SD-GCEP, providing the community with outcomes and updates from the group.

## Materials and Methods

### Membership

The membership of the SD-GCEP is composed of medical geneticists, genetic counselors, clinical molecular geneticists, variant scientists, and basic scientists, as well as staff biocurators from ClinGen. These members are largely volunteers from academic institutes, clinical laboratories, and organizations that provide online gene-level resources. Initial membership was solicited through invitation or self-nomination. New members can volunteer through ClinGen’s Community Curation Database (https://ccdb.clinicalgenome.org/apply) or by reaching out to the SD-GCEP coordinator directly (https://clinicalgenome.org/affiliation/40060).

### Identifying relevant genes

Five approaches have been used during the existence of the SD-GCEP to prioritize relevant GDRs for curation. Approach 1 was to identify syndromic GDRs most frequently tested in clinical laboratories, prioritized by the number of tests in the Genetic Testing Registry^10^ and the number of pathogenic or likely pathogenic variants in the gene in ClinVar. Approach 2 identified GDRs detected through clinical genome or exome sequencing performed by diagnostic laboratories within the membership of the SD-GCEP, utilizing and building upon the curation efforts performed as part of this testing. Approach 3 identified new GDRs from research consortia including National Human Genome Research Institute’s Centers of Mendelian Genomics and Genomics Research to Elucidate the Genetics of Rare diseases consortium, again utilizing existing internal efforts. Approach 4 included any GDRs requested by other GCEPs for phenotypes requiring the broad expertise of the SD-GCEP. Most recently, Approach 5 was used to capture additional syndromic GDRs that have been curated by groups not using the ClinGen framework by first searching the Gene Curation Coalition (GenCC) database (https://search.thegencc.org/) for disease assertions with “syndrome” in name, then prioritizing Strong or Definitive classifications given these are often included on gene panels. The genes from all approaches were reviewed to ensure the disease assertions were syndromic in nature, rather than pertaining to a single organ system, and the final list was reviewed and approved by the SD-GCEP chairs.

### Precuration for genes with multiple disease assertions

ClinGen’s precuration is the process of evaluating available information for GDRs to determine the disease entity and mode of inheritance to be curated.^11^ When evaluating genes with multiple disease assertions listed within ontologies such as OMIM,^12^ Orphanet,^13^ Mondo Disease Ontology (Mondo),^14^ or the literature, the SD-GCEP refers to ClinGen’s Lumping and Splitting guidelines^15^ to precurate the gene. Lumping involves combining 2 or more conditions into a single disease entity, whereas splitting involves separating disease assertions into distinct entities. To inform this decision, the molecular mechanism, phenotypic variability within and across families, and the mode of inheritance are reviewed for each disease assertion, and the decision to lump or split is then voted on by the expert panel. Following ClinGen’s guidelines, disorders are generally lumped into a single entity when the underlying molecular mechanism is consistent, and the clinical features represent a spectrum of the same condition. When there are insufficient data on the molecular mechanism or uncertainty on the phenotypic spectrum, disadvantages of splitting include dilution of evidence across multiple GDRs and underweighting of the final curation strength. The expert panel decides on the most appropriate name for the entity to be curated by applying the dyadic naming convention(s) for lumped conditions, which are defined by the ClinGen Guidance and Recommendations for Monogenic Disease Nomenclature.^16^ For example, for the curation of *SOX3*, the group voted to lump the disease assertions given in OMIM (ie, intellectual developmental disorder, X-linked, with isolated growth hormone deficiency and X-linked panhypopituitarism into 1 disorder under the new name “*SOX3*-related X-linked pituitary hormone deficiency with or without intellectual developmental disorder”). The naming decisions are communicated to OMIM, Mondo, and Orphanet for consideration of inclusion in ontology.

### Curation and expert panel review

The SD-GCEP evaluates the clinical validity of GDRs according to ClinGen’s gene curation workflow,^9^ and curation is performed by SD-GCEP curators using ClinGen’s Gene Curation Interface.^17^ Genetic and experimental data, either supporting or disputing the validity of the disease relationship, are examined and classified using the current standard operating procedures document (for this study, versions 7 to 10 depending on the date of curation [https://clinicalgenome.org/docs/?doc-type=curation-activity-procedures&curation-procedure=gene-disease-validity]) by a SD-GCEP biocurator. On average, 2 biocurators each present 1 curation per meeting on either the biweekly or quarterly video conference call. The final decision is voted on and approved by the members in attendance at the meeting, including both biocurators and experts. All members are eligible to vote except anyone with a conflict of interest (as reported to ClinGen annually) on a specific gene or disease who abstains. There is no specific quorum for voting purposes, although the chairs (with input from the members) monitor for any close votes or meetings with less than average attendance and assess whether further input is needed or the curation needs to be brought back for discussion at another meeting. A “Biocurator Feedback” Google form is made available during every call for members to leave comments or questions about the curations. Additionally, attendees share additional relevant data for or thoughts concerning the curation through verbal discussion or written messages in the meeting chat. Disagreements in scoring or classification are generally resolved through further discussion with attendees from each perspective encouraged to participate. Occasionally if needed, and/or additional information is required to resolve disagreements, smaller meetings may be held offline, the conclusions of which are brought back to the main meeting for discussion and voting by the wider GCEP. Curators submit evidence summaries for final review and approval by the chairs; these are then published to the ClinGen website for broad community access.

### SD-GCEP-specific guidelines

Many GCEPs adopt internal modifications to the ClinGen Gene-Disease Validity Standard Operating Procedures in places where there is a degree of subjectivity. In addition to the formal guidelines and to help with consistency, the SD-GCEP has created a comprehensive “SD-GCEP Scoring Guidance and Specifications Document” and a short form that includes additional guidance and detailed information specific to the SD-GCEP, including where to look for data and how to score them. Of note, considering human genetic evidence, the SD-GCEP specifically downgrades loss-of-function variants for homozygosity and if consanguinity cannot be ruled out, because these individuals are likely have multiple homozygous variants due to runs of homozygosity, and it may be unclear which homozygous variants are causative^18^ (Table 1). Because of the rarity of many syndromic phenotypes, the number of unique pathogenic variants may be limited, the SD-GCEP allows scoring of multiple observations of the same variant when de novo occurrence is proven. Each observation is awarded the default score for the variant type, as well as the upgrade for de novo status. Recurrently occurring variants that present with a highly specific phenotype and are known to act via a dominant mechanism are upgraded (Table 1). In considering experimental evidence, nonhuman animal models are thoroughly evaluated to determine whether the phenotype appropriately recapitulates the syndromic human disease. Models that lack reproducibility of multiple human phenotypes are downgraded to 1 point, instead of the default 2 points (Table 1). Models in which a human disease variant has been knocked in are considered for the maximum 4 points. Morpholino-mediated knockdown models are downgraded to 1 point unless re-expression of the wild-type allele demonstrates rescue of the phenotype.^19^

**Table 1:** SD-GCEP scoring guidance

Scenario	Recommended Score	
Genetic Evidence		
	Predicted or Proven Null Variants	Other Variant Types
Variant observed in individual with expected phenotype for the disease	1.5 points (suggested ClinGen default points)	0.1 (suggested ClinGen default points)
Variant homozygosity and/or parental consanguinity	−0.5 points for each (2.5 or 2.0 points total for proband)	Score each variant at 0.05 (0.1 points total for proband)
De novo occurrence of variant (each recurrent de novo can be scored at full strength)	+0.5 points (suggested ClinGen upgrade)	+0.4 points (suggested ClinGen upgrade)
Recurrent AD variants with highly specific phenotype	+0.25 points	+0.15 points
Variant not predicted to cause a true loss of function	−1.0 points	N/A
Mechanism of disease is unknown or has not been properly established	−0.5 points	0.1 (suggested ClinGen default points)
Variant located in hotspot or functional domain	N/A	+0.15 points
Functional modeling of variants: can consider increasing score for structural and 3D modeling (but not for in silico predictors alone)	N/A	+0.15 points (if the modeling is for specific amino acids (eg, Cys) that are known to be important to protein structure)

Experimental Evidence		

Animal model recapitulates syndromic human disease	2 points (suggested ClinGen default points)	
Transgenic model that overexpresses variant of interest, with recapitulation of syndromic phenotype	+1 points	
Animal model where human pathogenic variant is knocked in, with recapitulation of syndromic phenotype	+2 points maximum	
Knockdown animal model, without demonstration of rescue by WT allele	−1 point	
Knockdown animal model, with rescue by nonhuman WT allele	−0.5 points	
Animal model that lacks reproducibility of multiple phenotypes associated with human disease	−1 point	

*3D*, 3 dimensional; *AD*, autosomal dominant; *Cys*, cysteine; *N/A*, not applicable; *SD-GCEP*, ClinGen Syndromic Disorders Gene Curation Expert Panel; *WT*, wild type.

## Results

### Assessing gene-disease validity across syndromic disorders

**Figure 1:**
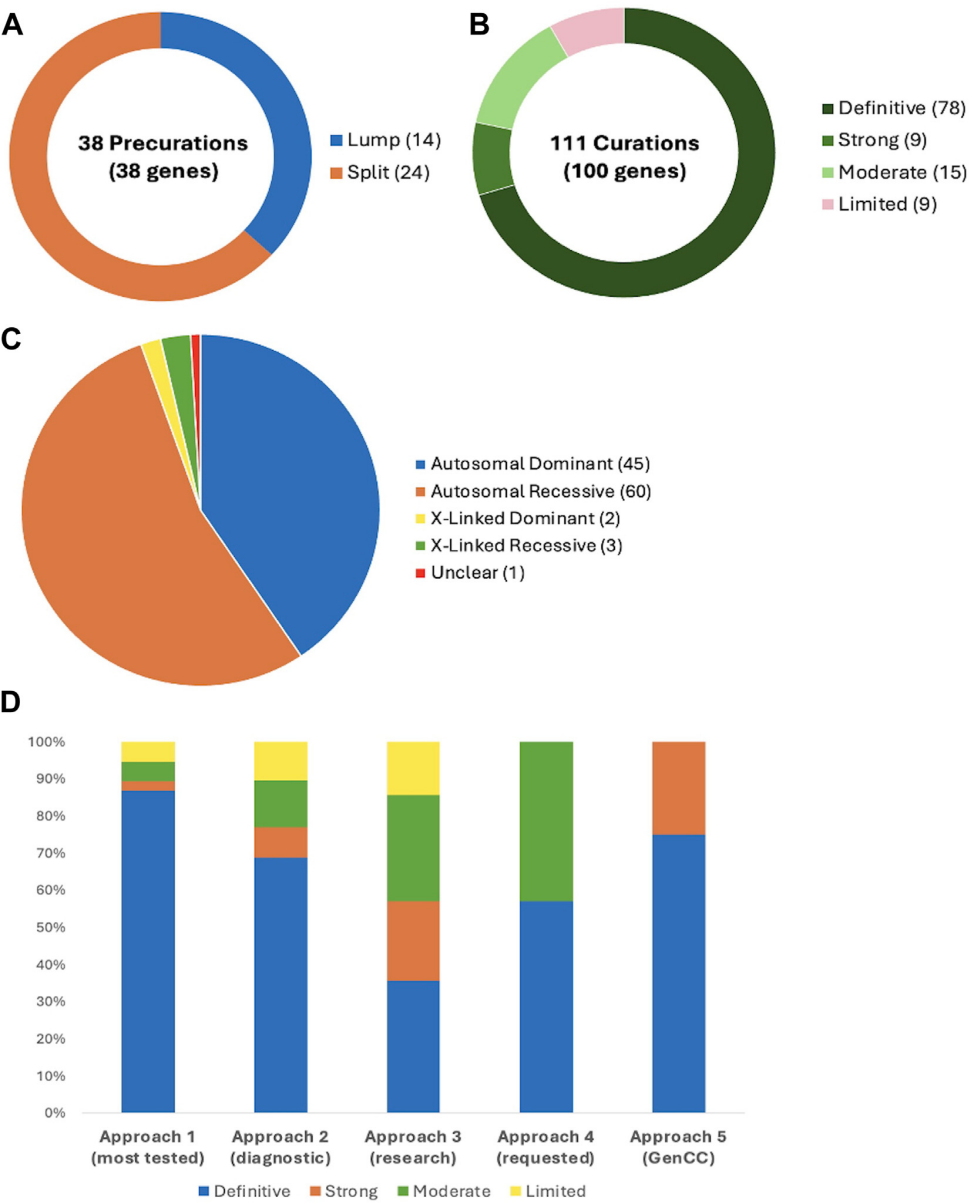
**Precurations and curations performed from April 2020 to March 2024.** A. Summary of lumping and splitting decisions for the 38 precurations performed to date. B. Summary of the final classifications for the 111 approved gene-disease relationship (GDR) classifications to date. C. Summary of mode of inheritance of the 111 approved GDRs to date. D. Summary of final classifications for GDRs across the 5 curation approaches. Approach 1 (38 genes: GDRs most frequently tested in clinical laboratories): 33 Definitive, 1 Strong, 2 Moderate, 2 Limited; Approach 2 (47 genes: GDRs through clinical genome or exome sequencing performed by diagnostic laboratories within the membership of the Syndromic Disorders Gene Curation Expert Panel [SD-GCEP]): 33 Definitive, 4 Strong, 6 Moderate, 5 Limited; Approach 3 (14 genes: new GDRs from research consortia, including National Human Genome Research Institute’s Centers of Mendelian Genomics and GREGoR consortium): 5 Definitive, 3 Strong, 4 Moderate, 2 Limited; Approach 4 (7 genes: GDRs requested by other GCEPs for phenotypes requiring the broad expertise of the SD-GCEP): 4 Definitive, 3 Moderate; Approach 5 (4 genes as of March 2024: syndromic GDRs in GenCC with Strong or Definitive classifications not curated by other GCEPs): 3 Definitive, 1 Strong. GenCC, Gene Curation Coalition; GREGoR, Genomics Research to Elucidate the Genetics of Rare diseases.

In the first 4 years, from April 2020 through March 2024, the SD-GCEP performed 38 precurations and 111 curations of GDRs involving 100 genes. For precurations, 14 genes with multiple assertions were lumped and curated as a single entity and 24 were split (Supplemental Table 1). For curations, 78 GDRs were classified as Definitive, 9 as Strong, 15 as Moderate, and 9 as Limited (Figures 1A and B, Supplemental Table 2). For the 111 GDRs, 60 had an autosomal recessive (AR) mode of inheritance, 45 were autosomal dominant, 3 were X-linked recessive, 2 were X-linked dominant, and 1 had an unclear mode of inheritance (Figure 1C). The unclear mode of inheritance is associated with the GDR for *UNC13A* and congenital nervous system disorder, which was curated by the SD-GCEP in 2021. At the time, 3 variants, including missense and stop-gained variants, had been reported in association with a neurodevelopmental syndrome characterized by variable features of developmental delay, seizures, microcephaly, and myopathy/movement disorders.^20^, ^21^, ^22^ Based on the limited number of reported cases, a distinct mode of inheritance, mechanism of disease, and phenotypic spectrum could not be determined, therefore the classification remains limited and provisional until further evidence is published.

**Figure 2:**
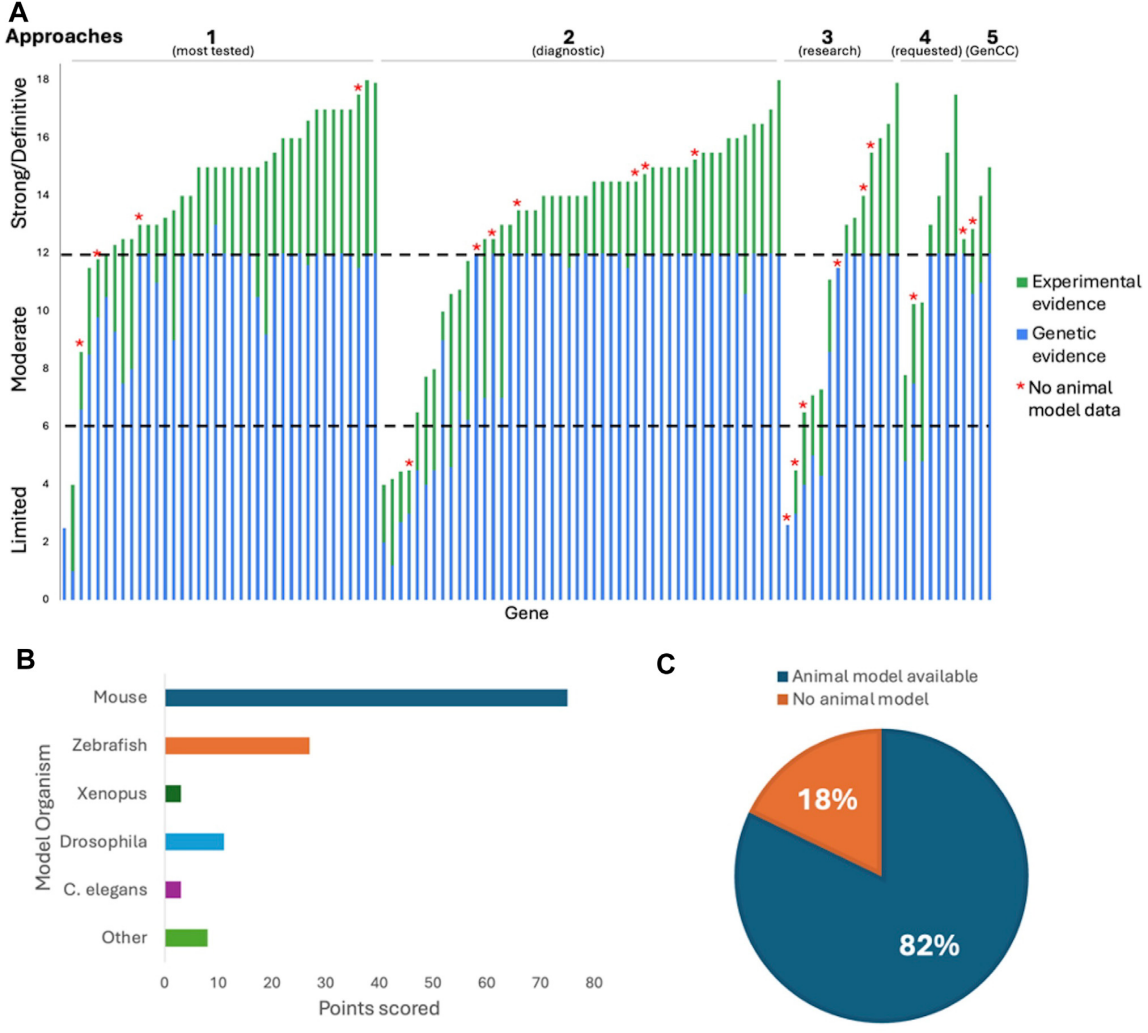
**Syndromic Disorders Gene Curation Expert Panel curation evidence.** A. The number of points awarded for genetic and experimental evidence for each gene represented by the bar height (blue = genetic evidence; green = experimental evidence). Gene-disease relationships (for which no animal model was available) are starred. B. The majority of genes (82%) had an animal model with 92% of sufficient quality and overlap with the human phenotype to be scored in the ClinGen curation framework. C. Mouse models were the predominant model organism scored. GenCC, Gene Curation Coalition.

The 38 GDR curations from the most frequently tested genes in clinical laboratories were more often classified as Definitive (“Approach 1”: 33 Definitive, 1 Strong, 2 Moderate, 2 Limited). This was similar to the distribution seen for the 48 GDR curations identified through clinical genomic sequencing performed by diagnostic laboratories by the membership of the SD-GCEP (“Approach 2”: 33 Definitive, 4 Strong, 6 Moderate, 5 Limited). A broader distribution was seen for the 14 GDR curations from within a research setting (“Approach 3”: 5 Definitive, 3 Strong, 4 Moderate, 2 Limited). Seven GDR curations came from collaboration with other GCEPs (“Approach 4”: 4 Definitive, 3 Moderate), and 4 GDR curations came from the more recently designed gene list looking at Strong and Definitive GDRs in the GenCC that have not yet been curated by ClinGen (“Approach 5”: 3 Definitive, 1 Strong) (Figure 1D). It was an open question whether GDRs without animal models would have sufficient evidence to reach classifications necessary to be included in diagnostic testing panels (Moderate or above); however, of the curations for which there were no animal models available to score, 8 were classified as Definitive, 4 as Strong, 4 as Moderate, and only 4 as Limited (Figure 2A). For example, the GDR for *ARSL* and X-linked chondrodysplasia punctata 1 is well defined in the literature, with variants in at least 50 probands in 7 publications scored in this curation,^23^, ^24^, ^25^, ^26^, ^27^, ^28^, ^29^ maxing out the genetic evidence at 12 points and reaching a Definitive classification, but it has no model organism. Overall, the presence of an animal model did correlate with the curation classification. 86.1% of curations classified as Definitive, 55.6% of curations classified as Strong, 66.7% of curations classified as Moderate, and 55.6% of curations classified as Limited had an animal model scored. Mouse models were the predominant model organism scored, whereas zebrafish, *Xenopus*, *Drosophila*, *Caenorhabditis elegans*, and other models have also been scored (Figure 2B). Eighty-two percent of all curations had animal models counted as experimental data (Figure 2C).

### Phenotypic diversity in syndromic disorders

**Figure 3:**
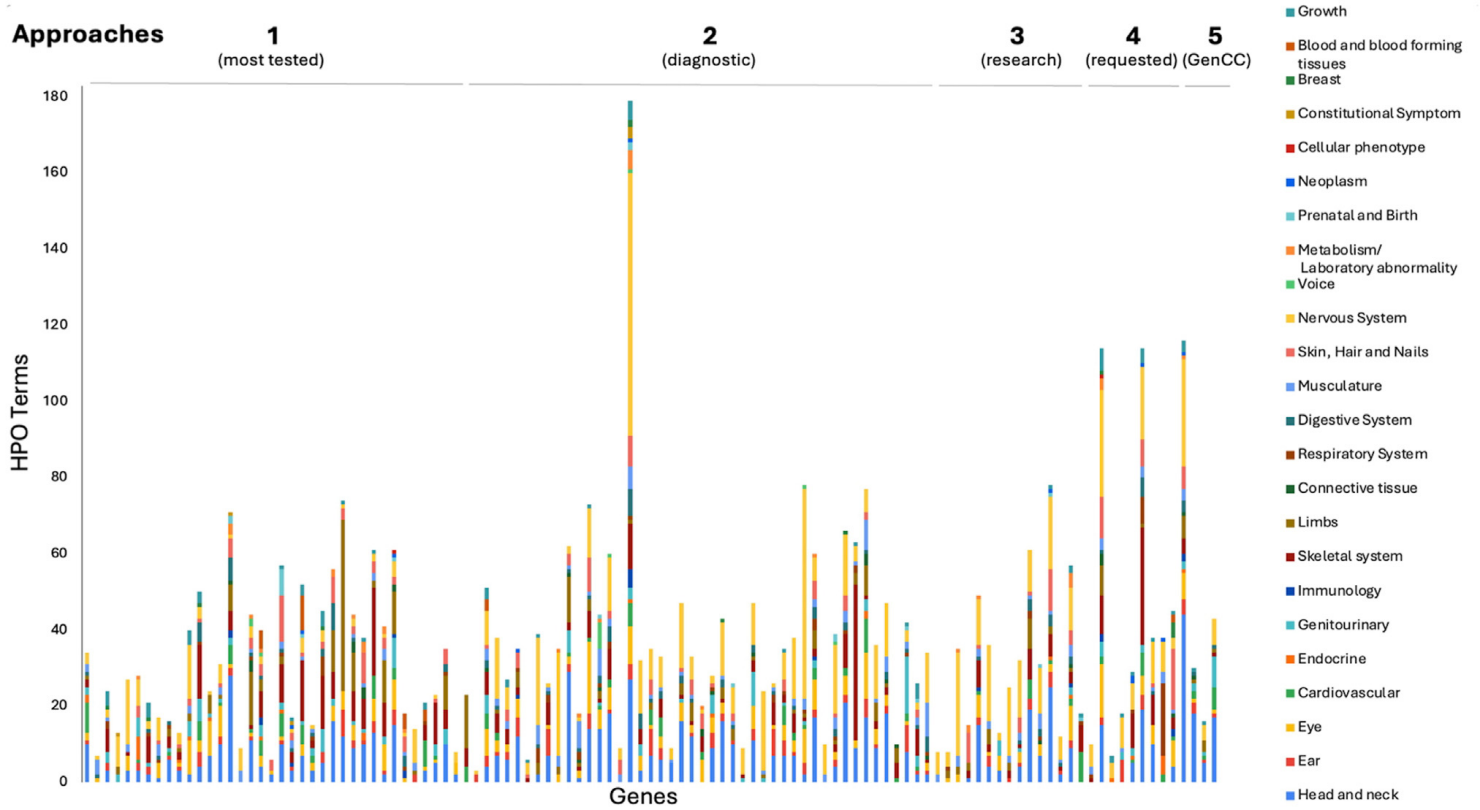
**Gene-disease relationship (GDRs) curated are highly syndromic in nature.** HPO higher order terms representing the phenotypic features associated with each disease assertion were graphed by counting the number of HPO terms under each higher order term. For terms that were lumped, HPO terms for all of the assertions were combined and counted. All diseases involved 2 or more organ systems, whereas the majority (88/111 gene-disease relationships, 79.2%) had 5 or more organ systems affected. GenCC, Gene Curation Coalition; HPO, Human Phenotype Ontology.

GDRs curated by the SD-GCEP are syndromic in nature, demonstrated by the diseases involved affecting more than 1 organ system. The number of Human Phenotype Ontology (HPO) terms under each of the 23 top-level terms by organ system (direct descendants of “Phenotypic abnormality, “HPO:0000118”) for each of the disease assertions for GDRs under curation was counted (Figure 3). All of the 23 different organ systems were affected in at least 1 of the curated GDRs. Of the 111 GDRs curated, a median of 8 organ systems were affected per disease. All diseases involved 2 or more organ systems, whereas the majority (88/111 GDRs, 79.2%) had 5 or more organ systems affected (Figure 3). The most commonly involved organ systems included the nervous system, head and neck, eye, and the skeletal system.

### Collaboration between GCEPs

**Figure 4:**
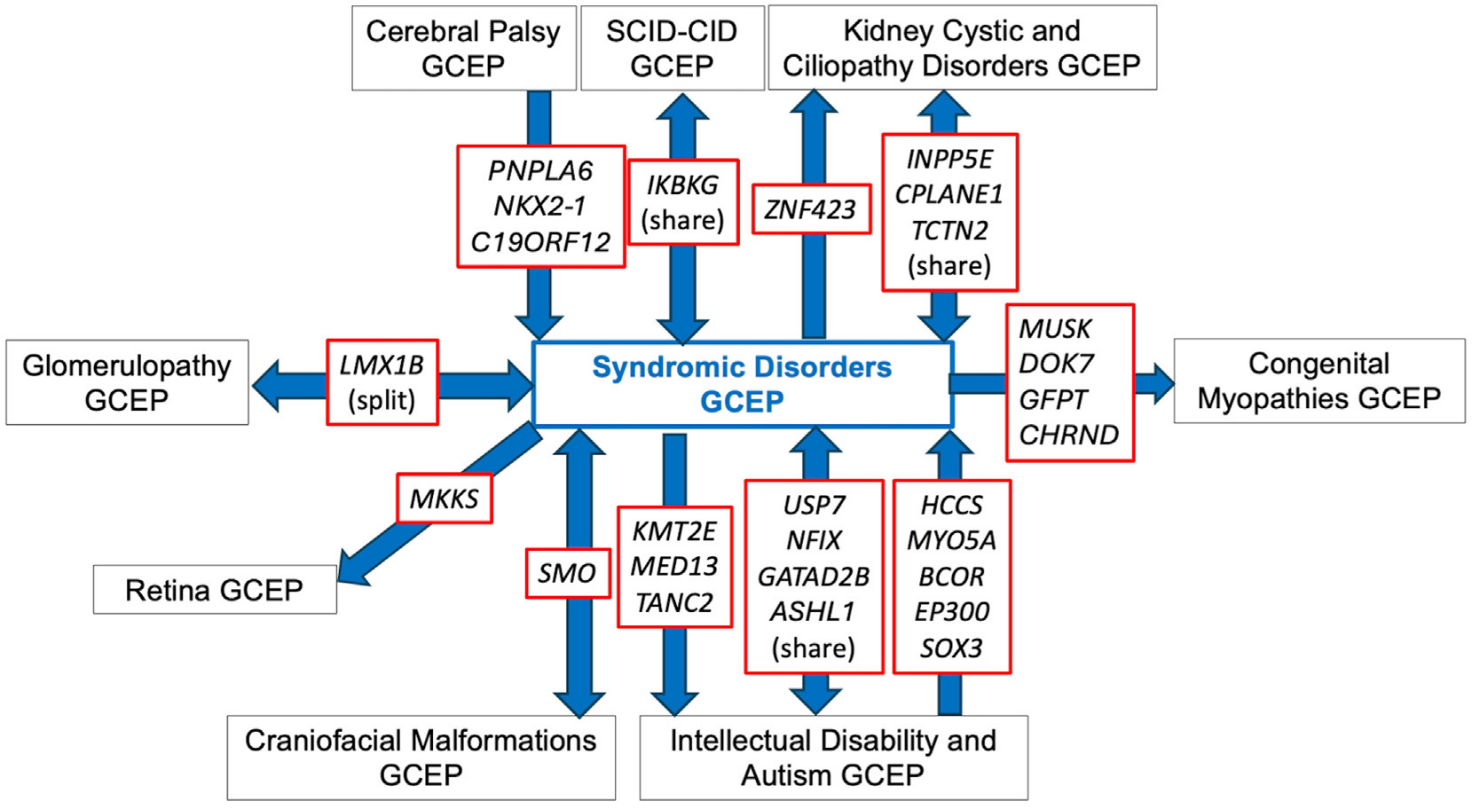
**Exchange and collaboration on gene-disease relationship curations between Syndromic Disorders Gene Curation Expert Panel (GCEP) and other GCEPs to date**.

The overlapping phenotypic features between syndromic disorders and other disease areas necessitated communication and collaboration between the SD-GCEP and other GCEPs. Genes are transferred to different GCEPs based on the specific expertise needed. For example, if a disease assertion is more syndromic than a GCEP is comfortable reviewing, they may transfer the gene to be curated by the SD-GCEP. Conversely, if a disease assertion originally thought to be syndromic mainly affects one organ system, it will be transferred to another GCEP if there is a specific GCEP for that disease area. Transfer to a more suitable GCEP also happened when new GCEPs were started after the SD-GCEP planned GDR lists were composed. Over the past 4 years, the SD-GCEP has collaborated and exchanged genes with 8 GCEPs: Cerebral Palsy, Congenital Myopathies, Craniofacial Malformations, Glomerulopathy, Intellectual Disability and Autism, Kidney Cystic and Ciliopathy Disorders, Retina, and Severe Combined Immune Deficiency and Combined Immune Deficiency GCEPs (Figure 4). Through this collaboration, 10 genes have been shared or split between the SD-GCEP and another GCEP (*USP7*, *NFIX*, *GATAD2B*, *ASHL1*, *SMO*, *LMX1B*, *IKBKG*, *INPP5E*, *CPLANE1*, and *TCTN2*). For example, the GDR *CPLANE1* and Joubert syndrome 17 was shared between the SD-GCEP from the Kidney Cystic and Ciliopathy Disorders (KCCD) GCEP because of overlapping phenotypes. The KCCD GCEP has curated several forms of Joubert syndrome because of the renal manifestations; however, patients with Joubert syndrome 17 and variants in *CPLANE1* were not found to have kidney involvement^30^; therefore, the SD-GCEP and KCCD GCEPs shared the curation and both are acknowledged as contributors on the ClinGen website.

Additionally, 9 genes have been transferred from the SD-GCEP to another GCEP (*KMT2E*, *MED13*, *TANC2*, *MKKS*, *ZNF423*, *MUSK*, *DOK7*, *GFPT*, and *CHRND*), and 8 genes have been transferred from another GCEP to the SD-GCEP (*HCCS*, *MYO5A*, *BCOR*, *EP300*, *SOX3*, *PNPLA6*, *NKX2-1*, and *C19ORF12*).

## Discussion

The SD-GCEP was created to address a gap in evaluating the clinical validity of GDRs involving multiple body systems and not under the purview of existing GCEPs. Additionally, the work of the SD-GCEP highlights the strength of the ClinGen framework. Syndromic disorders are individually rare and diverse in their presentations, and no single expert can be expected to have deep knowledge of every syndrome. In this setting, it is the expert-generalist approach with curators who are experienced in critical evaluation of evidence and application of the framework, combined with clinical and functional input from expert members with expertise in rare disease, which leads to accurate gene-disease classifications. Accurate GDR classifications are necessary for reporting because American College of Medical Genetics and Genomics recommends that diagnostic gene panels include all GDRs that meet criteria for Definitive, Strong, or Moderate evidence as defined by ClinGen.^6^

Robust and standardized gene curation is necessary for accurate and consistent clinical interpretation across all aspects of genomic sequence analysis, including variant triage, classification, and reporting decisions. The SD-GCEP has curated 102 GDRs with sufficient evidence to indicate clinical relevance (78 Definitive, 9 Strong, and 15 Moderate), supporting inclusion on diagnostic gene panels.^6^ As the composition of these panels changes over time to reflect current knowledge, reanalysis can yield additional diagnoses in approximately 10% to 20% of patients, the majority of which can be attributed to newly described GDRs.^31^^,^^32^ Variants with sufficient supporting evidence identified in genes with clinically relevant GDRs, are able to reach medically significant classifications of likely pathogenic or pathogenic, whereas those in genes with a Limited GDR should not be classified above variant of uncertain significance, which can affect return of results to patients depending on national or local guidelines. Numerous studies have detailed the direct impact of return of results from genomic testing by way of change to patient and/or family management including altered clinical management and reproductive decision making.^33^^,^^34^

The SD-GCEP also highlights the importance of collaborating with other GCEPs. These collaborations are necessary for the best-informed gene-disease classifications. For example, the Craniofacial Malformations GCEP performed a precuration on the gene *SMO* and recommended keeping the disease assertions for Curry-Jones syndrome and Pallister-Hall-like syndrome as split. Curry-Jones syndrome is a syndromic craniosynostosis; therefore, the Craniofacial Malformations GCEP curated that assertion but referred the Pallister-Hall-like syndrome assertion to be curated under the expertise of the SD-GCEP because it involves multiple body systems. Similarly, the SD-GCEP forwarded the *MKKS* gene to be curated under the expertise of the Retina GCEP because of the retinal phenotypes present. The SD-GCEP has also collaborated with the Primary Immune Regulatory Disorders GCEP, in which the Primary Immune Regulatory Disorders GCEP performed a secondary curation of *RAB27A* and Griscelli syndrome type 2, a rare AR disease characterized by cutaneous hypopigmentation, immunodeficiency, and hemophagocytic lymphohistiocytosis,^35^ adding additional phenotypic information on immunodeficiency to the evidence summary. Effective communication and collaboration between GCEPs is optimal for accurate gene-disease classifications.

Lumping multiple disease assertions after a precuration adds to the challenge of naming the curated disease entity. Rare diseases, including many syndromic disorders, are commonly named after clinicians and patients. This approach does not provide information about the expected clinical features as a result of variation within the gene of interest. The SD-GCEP follows dyadic naming convention as defined by the ClinGen’s guidance and recommendations for monogenic disease nomenclature for GDRs that reach a classification of Moderate, Strong, or Definitive.^16^ The new label for the entity contains the Human Genome Organisation Gene Nomenclature Committee gene symbol with the related phenotype. Of the 14 lumped curations, 7 have required the creation of a new name and identifier for the disease entity. These discussions are held in collaboration with relevant ClinGen expert panels and OMIM and Mondo nomenclature expert members of the SD-GCEP. For example, there were 6 disease assertions associated with the gene *PNPLA6*. During the precuration, the GCEP decided to lump the assertions cerebellar ataxia and spastic paraplegia into the term “*PNPLA6*-related spastic paraplegia with or without ataxia” and lump the terms Boucher-Neuhauser syndrome, Gordon Holmes syndrome, Laurence-Moon syndrome, and Oliver-McFarlane syndrome into the term “retinal dystrophy-ataxia-pituitary hormone abnormality-hypogonadism syndrome.” The GCEP worked closely with Mondo to have these new terms created (MONDO:0100149, MONDO:0100155).

Conversely, splitting multiple disease assertions during the precuration phase poses a challenge at the variant curation level. Gene curation and variant curation are closely connected because the final clinical validity classification can affect the expected variant classification. One way that the GDR classification is affected is through splitting disease assertions into separate entities based on the present data. For the 38 genes associated with multiple conditions precurated by the SD-GCEP, 66% (25/38) have been curated as split entities with 2 or more separate entities. For example, the *ENPP1* gene has 3 gene-disease assertions for arterial calcification generalized of infancy 1; hypophosphatemic rickets AR 2; and hypopigmentation-punctate palmoplantar keratoderma syndrome or Cole disease. A precuration identified that biallelic loss of function is the underlying mechanism of pathogenicity for all entities. However, based on the differences in inheritance pattern and clinical phenotype found in hypopigmentation-punctate palmoplantar keratoderma syndrome, the SD-GCEP decided to split this entity and lump arterial calcification generalized of infancy 1 and hypophosphatemic rickets AR 2 as 1 disease entity. The final clinical validity classification for the lumped entity was Definitive, whereas the split entity only reached Limited classification. Although splitting is justified utilizing a framework that examines the molecular mechanism, phenotypic spectrum, and inheritance patterns of the asserted disease entities, it may result in a reduction in the final evidence scores because the case reports and functional evidence are restricted to a specific condition rather than a broader lumped entity.^15^ If the final classification is Limited, No evidence, Disputed, or Refuted, it can reflect a lack of sufficient evidence supporting the syndrome, therefore potentially reducing the diagnosis rate.^6^ The SD-GCEP recognizes this limitation and plans to reconcile the problem by reevaluating these GDRs as defined by the ClinGen Standard Gene-Disease Relationship Recuration Procedure.^36^ Reevaluation of these curations every 2 to 3 years allows the consideration of new data and the possibility of upgrading the classification.

Another limitation the SD-GCEP faces is the lack of available evidence for extremely rare syndromes. When curating genes that are associated with extremely rare syndromes, the SD-GCEP has struggled with the limited number of cases to count toward genetic evidence. Curators are encouraged to explore publicly available databases such as ClinVar and DECIPHER for probands not in the published literature to avoid underscoring the case-level evidence. Likewise, lack of experimental and functional assays is also a common limiting factor in curation. Publication of case series and reports and of well-controlled experimental and functional assays remains critically important, particularly for extremely rare syndromes toward accurately curating GDRs.

Moving forward, the ClinGen SD-GCEP will continue to evaluate the clinical validity of GDRs, prioritizing genes listed as Strong or Definitive for a syndrome in GenCC and those recommended by our expert panel members. The GCEP will begin recurating genes previously classified as Strong, Moderate, or Limited while continuing to collaborate with other relevant GCEPs. By defining the clinical validity of GDRs involved in syndromic disorders, the SD-GCEP enables the incorporation of more clinically relevant genes in genetic testing panels and provides a critical resource to the community to improve diagnostic rates and patient outcomes.

## Data Availability

The data used in this publication are available in the Supplemental File and the online version of this article. The Clinical Genome Resource Syndromic Disorders Gene Curation Expert Panel makes all curations publicly available on the Clinical Genome Resource website (https://search.clinicalgenome.org/kb/gene-validity/).

## Conflict of Interest

Krista Bluske, Matthew P. Brown, Amanda Buchanan, Brendan T. Burns, Nicole J. Burns, Anjana Chandrasekhar, Aditi Chawla, Amanda R. Clause, Katie L. Golden-Grant, Akanchha Kesari, Alka Malhotra, Revathi Rajkumar, Zinayida Schlachetzki, Julie P. Taylor, Alison J. Coffey are current or former employees and shareholders of Illumina Inc. Krista Bluske, Jennifer M. Huang, Devon L. Thrush, Bess Wayburn are employees of Ambry Genetics. Katie L. Golden-Grant is an employee of Rady Children’s Institute for Genomic Medicine. Saumya S. Jamuar is the cofounder of Global Gene Corporation Pte Ltd. Julie P. Taylor is an employee of Blueprint Genetics (a Quest company). Anne O’Donnell-Luria was a paid consultant for Tome Biosciences, Ono Pharma USA, and Addition Therapeutics and received research funding from Pacific Biosciences. All other authors declare no conflicts of interest.

## References

[1] Christianson A., Howson C.P., Modell B. March of dimes global report on birth defects. March of Dimes Birth Defects Foundation. https://www.prevencioncongenitas.org/wp-content/uploads/2017/02/Global-report-on-birth-defects-The-hidden-toll-of-dying-and-disabled-children-Full-report.pdf.

[2] Mathews T.J., Driscoll A.K. (2017).

[3] Chung C.C.Y., Ng N.Y.T., Ng Y.N.C. (2023). Socio-economic costs of rare diseases and the risk of financial hardship: a cross-sectional study. Lancet Reg Health West Pac.

[4] Bamshad M.J., Nickerson D.A., Chong J.X. (2019). Mendelian gene discovery: fast and furious with no end in sight. Am J Hum Genet.

[5] Clause A.R., Taylor J.P., Rajkumar R. (2023). Reactive gene curation to support interpretation and reporting of a clinical genome test for rare disease: experience from over 1,000 cases. Cell Genom.

[6] Bean L.J.H., Funke B., Carlston C.M. (2020). Diagnostic gene sequencing panels: from design to report-a technical standard of the American College of Medical Genetics and Genomics (ACMG). Genet Med.

[7] Richards S., Aziz N., Bale S. (2015). Standards and guidelines for the interpretation of sequence variants: a joint consensus recommendation of the American College of Medical Genetics and Genomics and the Association for Molecular Pathology. Genet Med.

[8] Rehm H.L., Berg J.S., Brooks L.D. (2015). ClinGen—the clinical genome resource. N Engl J Med.

[9] Strande N.T., Riggs E.R., Buchanan A.H. (2017). Evaluating the clinical validity of gene-disease associations: an evidence-based framework developed by the Clinical Genome Resource. Am J Hum Genet.

[10] Rubinstein W.S., Maglott D.R., Lee J.M. (2013). The NIH genetic testing registry: a new, centralized database of genetic tests to enable access to comprehensive information and improve transparency. Nucleic Acids Res.

[11] Gene-disease validity training materials – ClinGen Clinical Genome Resource. https://clinicalgenome.org/curation-activities/gene-disease-validity/training-materials/.

[12] Hamosh A., Scott A.F., Amberger J.S., Bocchini C.A., McKusick V.A. (2005). Online Mendelian Inheritance in Man (OMIM), a knowledge base of human genes and genetic disorders. Nucleic Acids Res.

[13] Nguengang Wakap S., Lambert D.M., Olry A. (2020). Estimating cumulative point prevalence of rare diseases: analysis of the Orphanet database. Eur J Hum Genet.

[14] Home Ontology Lookup Service. https://www.ebi.ac.uk/ols4/.

[15] Thaxton C., Goldstein J., DiStefano M. (2022). Lumping versus splitting: how to approach defining a disease to enable accurate genomic curation. Cell Genom.

[16] Thaxton C., Biesecker L.G., DiStefano M. (2024). Implementation of a dyadic nomenclature for monogenic diseases. Am J Hum Genet.

[17] Wright M.W., Thaxton C.L., Nelson T. (2024). Generating clinical-grade gene-Disease Validity classifications through the ClinGen data platforms. Annu Rev Biomed Data Sci.

[18] Gene-disease validity curation process standard operating procedure, version 11. The Clinical Genome Resource Gene Curation Working Group. https://clinicalgenome.org/site/assets/files/9851/gene-disease_validity_standard_operating_procedures-_version_11_docx.pdf.

[19] Eisen J.S., Smith J.C. (2008). Controlling morpholino experiments: don’t stop making antisense. Development.

[20] Engel A.G., Selcen D., Shen X.M., Milone M., Harper C.M. (2016). Loss of MUNC13-1 function causes microcephaly, cortical hyperexcitability, and fatal myasthenia. Neurol Genet.

[21] Lipstein N., Verhoeven-Duif N.M., Michelassi F.E. (2017). Synaptic UNC13A protein variant causes increased neurotransmission and dyskinetic movement disorder. J Clin Invest.

[22] Lionel A.C., Costain G., Monfared N. (2018). Improved diagnostic yield compared with targeted gene sequencing panels suggests a role for whole-genome sequencing as a first-tier genetic test. Genet Med.

[23] Sheffield L.J., Osborn A.H., Hutchison W.M. (1998). Segregation of mutations in arylsulphatase E and correlation with the clinical presentation of chondrodysplasia punctata. J Med Genet.

[24] Parenti G., Buttitta P., Meroni G. (1997). X-linked recessive chondrodysplasia punctata due to a new point mutation of the ARSE gene. Am J Med Genet.

[25] Garnier A., Dauger S., Eurin D. (2007). Brachytelephalangic chondrodysplasia punctata with severe spinal cord compression: report of four new cases. Eur J Pediatr.

[26] Nino M., Matos-Miranda C., Maeda M. (2008). Clinical and molecular analysis of arylsulfatase E in patients with brachytelephalangic chondrodysplasia punctata. Am J Med Genet A.

[27] Franco B., Meroni G., Parenti G. (1995). A cluster of sulfatase genes on Xp22.3: mutations in chondrodysplasia punctata (CDPX) and implications for warfarin embryopathy. Cell.

[28] Brunetti-Pierri N., Andreucci M.V., Tuzzi R. (2003). X-linked recessive chondrodysplasia punctata: spectrum of arylsulfatase E gene mutations and expanded clinical variability. Am J Med Genet A.

[29] Matos-Miranda C., Nimmo G., Williams B. (2013). A prospective study of brachytelephalangic chondrodysplasia punctata: identification of arylsulfatase E mutations, functional analysis of novel missense alleles, and determination of potential phenocopies. Genet Med.

[30] Srour M., Schwartzentruber J., Hamdan F.F. (2012). Mutations in C5ORF42 cause Joubert syndrome in the French Canadian population. Am J Hum Genet.

[31] Wright C.F., McRae J.F., Clayton S. (2018). Making new genetic diagnoses with old data: iterative reanalysis and reporting from genome-wide data in 1,133 families with developmental disorders. Genet Med.

[32] van Slobbe M., van Haeringen A., Vissers L.E.L.M. (2024). Reanalysis of whole-exome sequencing (WES) data of children with neurodevelopmental disorders in a standard patient care context. Eur J Pediatr.

[33] Malinowski J., Miller D.T., Demmer L. (2020). Systematic evidence-based review: outcomes from exome and genome sequencing for pediatric patients with congenital anomalies or intellectual disability. Genet Med.

[34] Krantz I.D., Medne L., NICUSeq Study Group (2021). Effect of whole-genome sequencing on the clinical management of acutely ill infants with suspected genetic disease: a randomized clinical trial. JAMA Pediatr.

[35] Mishra K., Singla S., Sharma S., Saxena R., Batra V.V. (2014). Griscelli syndrome type 2: a novel mutation in RAB27A gene with different clinical features in 2 siblings: a diagnostic conundrum. Korean J Pediatr.

[36] Standard gene-disease relationship Recuration procedure(s). Clinical Genome Resource. https://www.clinicalgenome.org/site/assets/files/2164/clingen_standard_gene-disease_validity_recuration_procedures_v1.pdf.

